# Supplementary material for: Impact of κ-Carrageenan on the Cold-Set Pea Protein Isolate Emulsion-Filled Gels: Mechanical Property, Microstructure, and In Vitro Digestive Behavior
Source: Foods. 2024 Feb 2;13(3):483. doi: 10.3390/foods13030483 (PMC10855759; doi:10.3390/foods13030483)
Supplement: Supplementary file 1 [file foods-13-00483-s001.zip › foods-2841643-supplementary.pdf]

Supplementary Data

Impact of κ-carrageenan on the cold-set pea protein isolate emulsion-filled gels: Mechanical property, microstructure, and *in vitro* digestive behavior

Xiaojiao Li[1,2], Xing Chen[1,2**], Hao Cheng[1,2*]

[1]State Key Laboratory of Food Science and Resources, Jiangnan University, Wuxi, Jiangsu, China

[2]School of Food Science and Technology, Jiangnan University, Wuxi, Jiangsu, China

***Corresponding author 1: Hao Cheng**

Address: State Key Laboratory of Food Science and Resources, School of Food Science and Technology, Jiangnan University, Wuxi, Jiangsu 214122, China.

Telephone: +86(510)8519-7367;

Email: haocheng@jiangnan.edu.cn

****Corresponding author 2: Xing Chen**

Address: State Key Laboratory of Food Science and Resources, School of Food Science and Technology, Jiangnan University, Wuxi, Jiangsu 214122, China.

Telephone: +86(510)8519-7367;

Email: xingchen@jiangnan.edu.cn

Figure S1

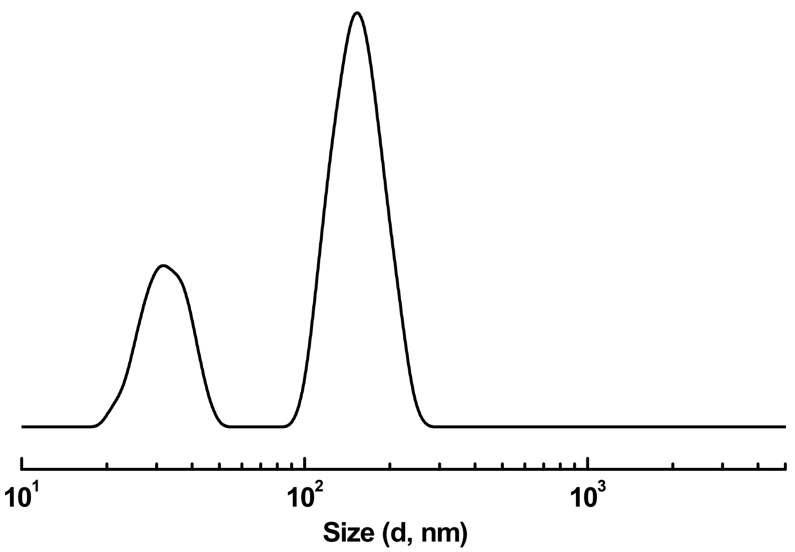

Fig. S1 Size distribution of pea protein isolate aggregates.
